# Development and Optimization of Solanum Lycocarpum Polyphenol Oxidase-Based Biosensor and Application towards Paracetamol Detection

**DOI:** 10.34172/apb.2021.054

**Published:** 2020-07-07

**Authors:** Rafael Souza Antunes, Douglas Vieira Thomaz, Luane Ferreira Garcia, Eric de Souza Gil, Flavio Marques Lopes

**Affiliations:** Faculty of Pharmacy, Federal University of Goias, R. 240, S/n - Setor Leste Universitário, Goiânia - GO, Brazil. Zip Code: 74605-170.

**Keywords:** Phenolic drugs, Biosensing technology, Enzymatic extract, Carbon paste electrode, Vegetal enzyme

## Abstract

***Purpose:*** The development biosensing technologies capable of delivering fast and reliable analysis is a growing trend in drug quality control. Considering the emerging use of plant-based polyphenol oxidases (PPO) as biological component of electrochemical biosensors, this work reports the first *Solanum lycocarpum* PPO biosensor and its use in the pharmaceutical analysis of paracetamol in tablet formulations.

***Methods:*** The biosensor was optimized regarding fruit maturation (immature and mature-ripe), vegetal extract volume to be used in biosensor construction as well as optimal pH of electrochemical cell fluid.

***Results:*** Results evidenced that the extract which rendered the biosensor with best analytical performance was from immature fruits, and the biosensor produced using 100 µL of crude plant extract promoted better faradaic signal gathering. Moreover, when neutral pH media was used in the electrochemical cell, the biosensor showcased best faradaic signal output from the used redox probe (catechol), suggesting thence that the method presents high sensibility for phenolic compounds detection. Furthermore, the biosensor was able to quantify paracetamol in a linear range from 50 to 300 μM, showcasing LoD and LoQ of 3 μM and 10 μM, respectively.

***Conclusion:*** after careful evaluation, this biosensor might be a low-cost alternative for conventional pharmaceutical quality control methods.

## Introduction

Innovative biotechnological approaches for drug quality control are highly regarded due to the possibility of reducing overall cost of assays and promoting environmental-friendly alternatives to traditional analytic techniques.^1,2^ Amongst the most recent advances in this field are biosensing technologies, which associate the well-known selectivity and sensibility of biochemical catalysis to analytical transduction.^3^ Although literature reports several transduction systems in the development of methods for pharmaceutical analysis, electrochemical transduction is considered a reliable and low-cost way to comprehensively investigate the amount of drugs in drug formulations.^3,4^

Electroanalytical methods such as voltammetry are remarkable in the investigation of the kinetics and thermodynamics of redox processes occurring at electrode surface.^5^ In this sense, the surface modification of electrodes through biological components may provide better analytical features such as lower detection and quantification limits.^6^ Moreover, electrode materials of high surface area such as carbon paste may be associated to these modifications to further enhance analytical features.^7,8^

Regarding the most used materials in electrode surface modifications aiming the improvement of pharmaceutical quality control methods, vegetal enzymatic extracts which are rich in polyphenol oxidase enzymes (PPOs) are highly regarded for their remarkable low cost and easy obtainable nature. Nonetheless literature reports the use of crude extracts as well as PPO-based enzymatic extracts from several vegetal sources in the development of biosensors intended to be used in pharmaceutical analysis of phenolic drugs such as paracetamol and methyldopa.^9,10^ Although many plant materials are known to render enzymatically rich extracts which may find use in biosensor development, previous reports evidenced the correlation between protein content and the enzymatic activity of vegetal extracts, what therefore suggests that plant developmental stage may play an important role in the optimization of a biosensing device.^9–11^

Amongst PPO-rich vegetal sources are Solanaceae family members such as *Solanum lycocarpum*. The highly active phenylpropanoid metabolism of this plant renders extracts of remarkable PPO yield and activity.^12^ *S. lycocarpum* is a common vegetal in Brazilian Cerrado, and is considered a trademark plant of this region, what therefore leads to its incorporation in local culinary and folk medicine.^13,14^ Although well known by Brazilian population, *S. lycocarpum* biotechnological potential is still untamed, what evidences the importance of further investigating its applicability in industrial contexts.^15,16^

Therefore, the aim of this study is to develop and optimize a *S. lycocarpum*-based biosensor and investigate its stability and reliability regarding the analytical detection of phenolic compounds. In this context, enzymatic extracts were prepared from immature and mature-ripe fruits and investigated regarding their protein content, PPO activity and their application in the development of biosensors for the quality control of phenolic drugs.

## Materials and Methods

### Reagents and solutions

All solutions were prepared using analytical-grade salts from Vetec Química Fina Ltda. (Rio de Janeiro, Brazil). Moreover, all aqueous solutions were prepared with ultrapure water of Millipore Milli-Q quality (conductivity ≤ 0.1 µS cm-^1^) (Molsheim, França). Paracetamol standard was acquired from Sigma-Aldrich (St. Louis, MO, USA). Standard stock solutions were prepared to render a concentration of 1 mM, from which work solutions of 100 µM were prepared.

### Vegetal material and crude extract

*Solanum lycocarpum* fruits were acquired from a single plant located in Anápolis-GO, Brazil (16°19’36” S; 48°57’10” W). All samplings took place in June 2017. Two mature-ripe and two immature fruits were collected and subjected to surface-rinsing with water followed by storage at 4°C in polyethylene bags.

The crude vegetal extract was prepared by processing 30 g of the pericarps in 100 mL of phosphate buffer solution (PBS) 0.05 M (pH 6.0). The processing was conducted using a commercial blender (Britânia, Brasil) for 2 minutes. The resulting homogenized solution was filtrated in cloth tissue, and the filtrated was labeled and used as the crude extract for biosensor development. Each extract was tagged according to its source, namely: Lob_mat_.EE which stands for the extract prepared from mature-ripe fruits, and Lob.EE; which stands for the extract prepared from immature fruits. All experiments were conducted under controlled temperature of 20°C ± 2°C.

### PPO enzymatic activity and protein analysis

In order to evaluate PPO activity, 100 μL of extract (either Lob.EE or Lob_mat_.EE) was added to 3 mL of catechol solution at 0.07 M. The catechol solution was prepared with 0.05 M PBS, pH 6.0). After 10 min, absorbance was recorded at 420 nm using UV-visible spectrophotometer (Q798U2VS, Quimis Aparelhos Científicos Ltda., São Paulo, Brazil).^17^ PPO activity was expressed in U/mg of protein.

Total protein was determined by Bradford method,^18^ in which bovine serum albumin (BSA) was used as standard. Thence, 100 μL of either Lob.EE or Lob_mat_.EE were mixed with 5 mL of Bradford reagent, and after 10 min the absorbance was recorded at 595 nm. All assays were conducted in triplicates under controlled temperature of 20 ± 2°C.

### Biosensor development and optimization

The conductive material selected for this work was carbon, which was used to build carbon paste electrodes through agglutination with mineral oil. Both the carbon and mineral oil were purchased from Sigma-Aldrich (St. Louis, MO, USA). Literature reports the wide applicability of carbon paste-based electrodes for biosensor development due to facilitated adsorption of biologic material on graphene sheets surface.^8-10^ Therefore, the crude extract/enzymatic extract was added directly to carbon powder, followed by homogenization and drying at 20°C ± 2°C. Mineral oil was added thereafter, and the resulting carbon paste was fitted into an insulated nylon support (Ø = 1 mm) coupled to a conductive copper rod.

The first optimization step involved the investigation of which fruit developmental stage would render biosensors of higher sensibility. Therefore, each extract was used to build biosensors intended to detect a phenolic probe (i.e.catechol at 0.07 M in 0.05 M PBS, pH 6). The extract which displayed highest sensibility in the detection of the phenolic probe was selected for further optimizations.

The second optimization step regarding the biosensor development concerned the amount of extract to be added to the carbon powder. Therefore, volumes ranging from 50 µL to 200 µL were used to build biosensors intended to undergo investigation using catechol-probe at 0.07 M in 0.05 M PBS, pH 6. Table 1 showcases biosensor compositions used in this step.

**Table 1:** Raw carbon paste (CP) and *S. lycocarpum* PPO-based biosensor (CP-Lob) composition. CP-Lob was optimized according to extract volume

**Sensor or Biosensor**	**Carbon powder (mg)**	**Added extract volume (µL)**	**Mineral oil (mg)**
CP	100	-	30
CP-Lob 50	100	50	30
CP-Lob100	100	100	30
CP-Lob150	100	150	30
CP-Lob200	100	200	30

The third optimization step was focused on the optimal pH for the assays. Thus, catechol was again used as a phenolic probe after adjustment of the final solution pH value to either 3.0, 5.0, 7.0 or 9.0.

### Electrochemical analysis

Electrochemical analysis was conducted using cathodic scan in order to detect PPO-mediated oxidation products. Therefore, differential pulse voltammetry (DPV) was selected due to its sensibility regarding faradaic current determination. A PGSTAT^®^ potentiostat model 204 with FRA32M module (Metrohm Autolab) was integrated to NOVA 2.1^®^ software and used to conduct all assays. All measurements were performed in a 1 mL one-compartment electrochemical cell coupled to a three-electrode system consisting of the biosensor herein developed, Pt wire and Ag/AgCl/KClsat (both purchased from Lab solutions, São Paulo, Brazil). The electrodes cited above represent the working, counter and reference electrodes, respectively.

DPV analysis was conducted using pulse amplitude of 50 mV, pulse width of 0.5 s and scan rate of 10 mV s-^1^. Before each DPV assay, the biosensors were subjected to 10 cycles of cyclic voltammetry scans between 0 and 1.0 V at 100 mV s-^1^ in order to stabilize the signal. All voltammograms were baseline-corrected and the data was treated using Origin 8^®^ software (OriginLab Corporation, Northampton, MA, USA).

### Biosensor storage stability and reuse

The storage stability test was performed in a period of 15 days. In this test, six CP-Lob100 were made and stored at 4°C. In the interval of 1, 3, 6, 9, 12 and 15 days, consecutively, a paste was removed from the refrigerator and set at 20 ± 2°C. Soon after, it was used to fill the Teflon cylindrical electrode (Ø = 1 mm).

Biosensor reuse was investigated in the optimized biosensor, and 5 consecutive analysis were conducted without surface-renewal. Once again, biosensor response was investigated by catechol probe detection using DPV.

### Analytical study towards paracetamol determination

At first, the optimized biosensor was used to assay a paracetamol calibration curve at concentration interval of 50 to 300 μM. The limit of detection (LoD) was defined as the smallest paracetamol concentration which could be detected using the biosensor, while the limit of quantification (LoQ) was the smallest concentration which could be quantified. Thereafter, the biosensor was subjected to standard recovery test assuming a maximum error of 5%. All tests were conducted according to standard pharmacopoeic protocols, being both LoD and LoQ calculated in according to these protocols.^19^

The pharmaceutical determination of paracetamol was also conducted in commercial tablets, being the optimized biosensor used for the assays. Therefore, 10 paracetamol tablets of 750 mg/tablet were grounded and used to prepare 1mM paracetamol stock solution. Thereafter, aliquots from the stock solution were diluted up to 100 μM, being this concentration used in voltammetric assays. A standard pharmacopoeic spectrophotometric method for paracetamol determination was used in order to compare results, being the readings recorded at 257 nm.^20^

### Statistical analysis

Statistical analysis was conducted using BioEstat^®^ software, version 5.3. The statistical differences between groups were determined using Tukey`s test, and statistical significance was attributed to *P* < 0.05. Furthermore, Origin 8^®^ software was used to render graphical depictions of the voltammograms (OriginLab Corporation, Northampton, MA, USA).

## Results and Discussion

### PPO enzymatic activity and protein analysis

Results evidenced that the extract from immature fruits presented higher protein levels as well as PPO activity than the extract from mature-ripe fruits, being their levels of 1013 mg protein/100 µL and 612 U/mg protein for Lob.EE, and 879 mg protein/100 µL and 297 U/mg protein for Lob_mat_.EE. These findings are sound when compared to literature, which describes the biochemical modulation of plant metabolism during fruit ripening.^21^ It is well stated that immature fruits present more active protein metabolism than ripe fruits in order to fully develop pericarp features. Moreover, the PPO activity in immature fruits may be higher than in mature-ripe fruits given that the pericarp phenolic profile is reported to drastically change according to ripening, what may suggest that the higher PPO activity as well as protein content may play a role in these chemical changes during fruit development and senescence.^21-23^

### Biosensor development and optimization

**Figure 1:**
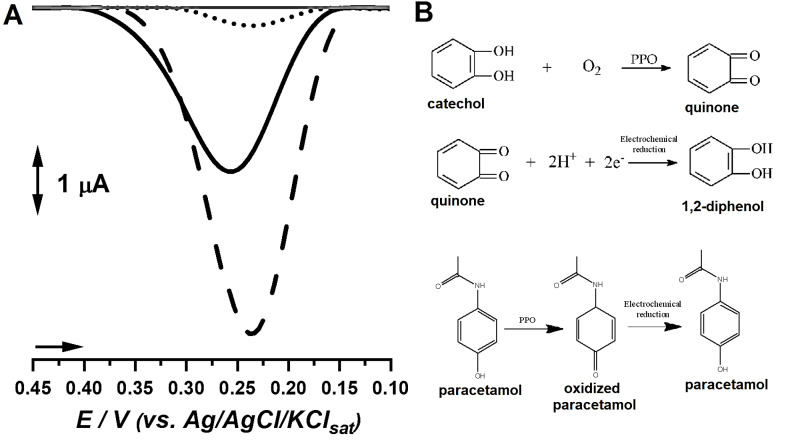
**(A)** DP voltammograms of catechol probe under analysis by bare carbon paste electrode (**●●●**), biosensor produced with the extract from mature-ripe *S. lycocarpum* fruits (▬) and from immature fruits (---). **(B)** Mechanism involved in the detection of catechol by the proposed biosensor and oxidative stages of paracetamol during analysis.

In order to evaluate the response of each biosensor for the detection of the phenolic probe, both the extract from immature fruits and mature-ripe fruits were used to detect catechol. Results are depicted in Figure 1.

Results showcased that the extract from immature fruits yielded a biosensor capable of providing higher analytical signal for catechol, followed by the biosensor built with the mature-ripe fruits, which increased catechol signal when compared to bare carbon paste. Bare carbon paste presented the smallest signal, what therefore suggests that the biosensors might provide better analytical features towards phenolic compounds detection (Figure 1).

The findings regarding catechol detection were further corroborated by protein and PPO investigations, which hinted beforehand that the extract from immature fruits may provide better analytical signals in enzyme-based tests. Moreover, several reports evidenced the correlation between PPO activity, protein content and analytic signal in biosensor development, and associated the better phenolic probe detection to PPO-mediated oxidation of phenolic moieties followed by electrochemical reduction of ketone residues, as showcased in Figure 1.^9-10,24,25^ In view of these findings, the biosensor produced from immature fruits (i.e. Lob.EE) was selected for further optimization steps.

**Figure 2:**
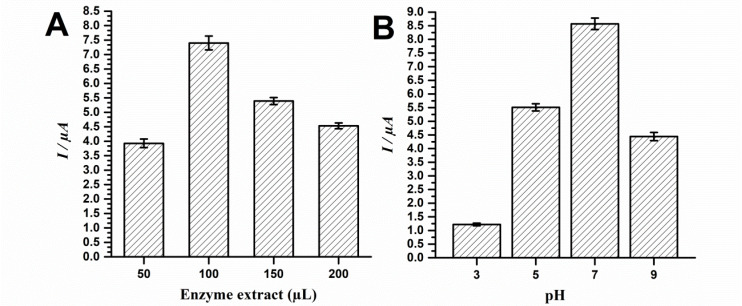
Optimization of the biosensor from immature *S. lycocarpum* fruits according to **(A)** added extract volume and **(B)** analytic solution pH.

The following optimization step involved the investigation of which extract volume would provide best phenolic probe detection. Therefore, several biosensors were built with varying Lob.EE volumes and tested for catechol detection. Furthermore, the biosensor was optimized regarding the best pH of the electrochemical cell fluid aiming higher detection of phenolic compounds. Results are displayed in Figure 2.

Results showcased that the best analytic signal for catechol detection was obtained using 100 µL of Lob.EE. Additional volumes seemingly hindered probe detection, therefore suggesting saturation (Figure 2A). Moreover, results evidenced that the biosensor presented best analytical performance at pH 7, followed by pH 5 and 9. The pH of 3 led to smallest catechol signal (Figure 2B).

The findings of the extract volume optimization step suggest that the excessive amount of biologic material may hinder analytical performance. Although results may seem contradictory at first, given that the extract with best protein content presented best probe detection features, the transduction system herein used is electrochemical, what therefore implies that insulating biomaterials such as proteins may hinder signal gathering. In this sense, given that the extracts were not subjected to further purification procedures, the excess of non-conducting molecules such as carbohydrates, lipids and oxidation products of secondary metabolites may impair probe detection in higher volumes such as 150 and 200 µL.^9,10,26,27^ Moreover, electrode fouling is a major concern in electroanalysis,^28,29^ and the minimization of these effects through optimizations is essential to ensure reproducibility.

The pH optimization evidenced that neutral pH leads to the best analytical performance, which may occur because PPOs may exert better catalytic effect under this condition.^30^ Moreover, given the pKa of phenols such as catechol, neutral pH may allow more molecules to be available to undergo biological catalysis, what thence enhances the analytic signal. Furthermore, excessively low or high pH values such as 3 or 9 may lead to enzyme denaturation, what therefore impairs proper biosensing.^30,31^

### Biosensor storage stability and reuse

**Figure 3:**
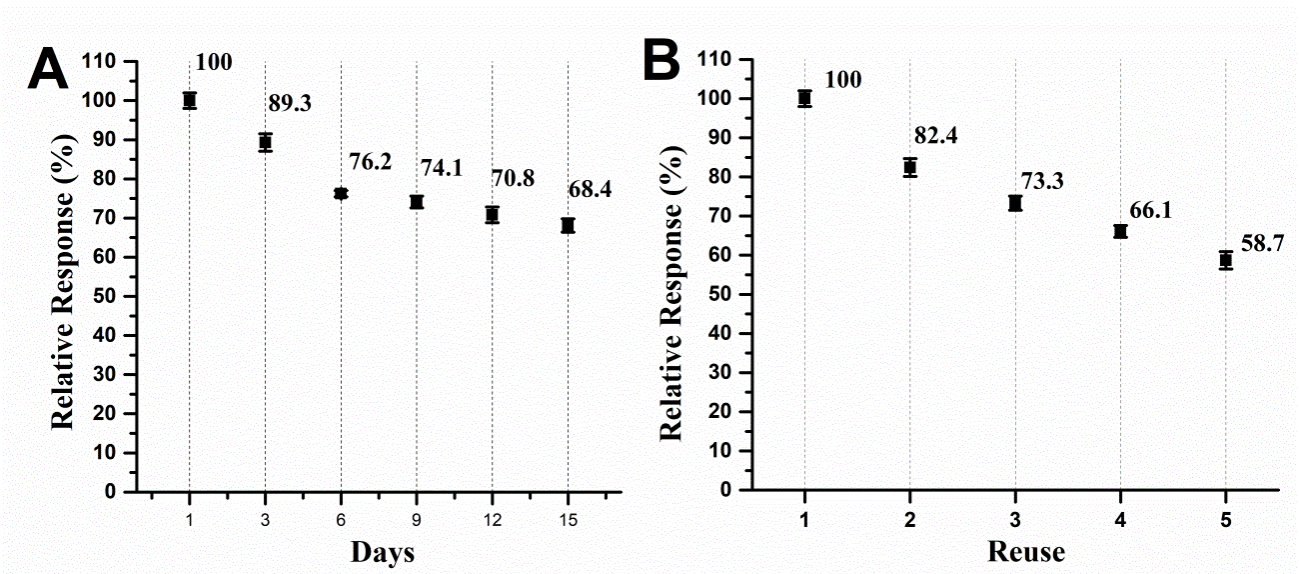
Storage stability and **(B)** reuse studies of the biosensor from immature *S. lycocarpum* fruits.

Biosensor storage stability and reuse were assayed in order to investigate if the analytical performance would significantly change over time, and if reuses of the biosensor could be achievable. Results are displayed in Figure 3.

**Figure 4:**
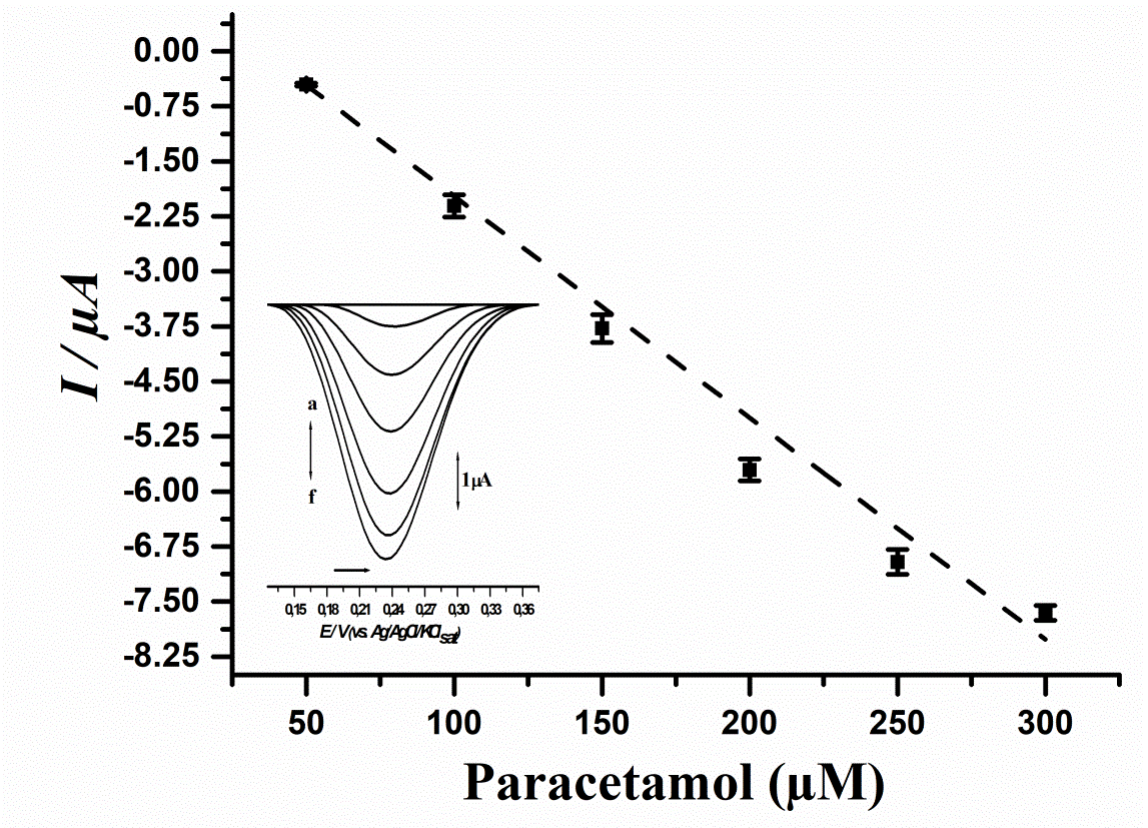
Analytical curve of paracetamol using the biosensor developed from immature *S. lycocarpum* fruits. Curve equation: I (µA) = 0.8058 – 0.031 [Paracetamol µM], r = 0.9918.

Results showcased that the biosensor lost analytical performance in a seemingly steady fashion over the course of storage time, what is a normal finding for biological material. The performance dropped about 10.7% at the third day and decreased until 68.4% of the former performance, what can be considered a remarkable finding given the simple, environmentally friendly and low-cost way in which the biologic material was immobilized in the CP (Figure 4A). Moreover, the reuse of the biosensor without surface renewal led to a drop in probe detection, which is a common finding in the electroanalysis of phenolic compounds,^28,29^ being the total detected signal after first assay of 82.4% and peaked 58.7% of the former performance in the fifth scan.

Stability is a major concern regarding bioproduct development due to the proneness of enzymes to undergo denaturation upon physicochemical shifts during their storage time. In this sense, most studies evidence that enzymatic extracts showcase decrease of the catalytic power after a few days of storage. The biosensor herein developed is stable in the first few post-production days, and given that it is designed for fast and reliable determination of phenolic compounds, results are remarkable.

The biosensor testing showcased results that allowed us to suggest that the adsorption/entrapment of the biological material on the carbon paste was seemingly effective when compared to complex chemo-adsorption methods, given the final performance of 68.4%, what is nonetheless noteworthy given the low-cost and environmentally friendly way in which the adsorption protocol was herein conducted.^29,32,33^

Concerning biosensor reuse, the electrode fouling effect of phenol-oxidation products is well described in literature and considered a major drawback in electroanalysis due to the formation of non-conductive polymers on electrode surface.^28,29,34-36^ However, considering the easy surface renewal of CP-based electrodes, this effect does not hinder their reliability, as reported previously.^9,10^

### Analytical study towards paracetamol determination

In order to evaluate the linearity of the biosensor method for paracetamol detection, a calibration curve was assayed. Results are showcased in Figure 4, while the comparison of the proposed method to up to date literature regarding paracetamol detection by biosensors is showcased in Table 2.

**Table 2:** Recovery test of paracetamol standard using the herein proposed biosensor (n = 3)

**Drug**	**Added concentration (μM)**	**Expected concentration (μM)**	Assay result (μM)*	Relative error (%)**	**Recovery (%)**
Paracetamol (100 μM)	0	100	101.17 ± 0.37	1.17	101.17
	50	150	146.09 ± 0.46	2.61	97.39
	100	200	202.03 ± 0.98	1.01	101.01
	200	300	294.26 ± 0.81	1.92	98.08

*Recovered concentrations according to the method herein developed. **Relative error between the assay results and the expected concentrations.

Results showcased that the biosensor method was linear to paracetamol concentrations ranging from 50 to 300 μM, and the estimated values of LoD and LoQ were respectively: 3 μM and 10 μM. Moreover, a recovery test was performed, and results are depicted in Table 3.

**Table 3:** PPO based biosensors for paracetamol pharmaceutical analysis

**PPO Plant Source**	**Linear Range (mM)**	LoQ (mM)*	LoD (mM)**	**References**
*Persea Americana*	1200 – 53000	880	880	^35^
*Solanum melongena*	20 - 200	20	5	^40^
*Solanum paniculatum*	5 - 245	5	3	^39^
*Genipa americana*	10 - 310	10	5	^10^
*Solanum lycocarpum*	50 – 300	10	3	Present work

* LoQ: quantification limit ** LoD: detection limit

Results evidenced that the biosensor presented good analytical parameters, with LoD and LoQ comparable to up-to-date literature regarding vegetal enzyme-based biosensors,^37-40^ as depicted in Table 3. Moreover, the recovery test showcased that the method is reproducible and reliable (Figure 4 and Table 3).

Furthermore, the biosensor was tested for the detection of paracetamol in pharmaceutical tablets and the results were compared to those of a standard spectrophotometric method. Results evidenced that the biosensor method quantified 744.08 ± 0.64 mg (i.e. 99.21%) while the standard method quantified 754.11 ± 1.42 mg (i.e. 100.55%) of 750 mg-labeled paracetamol tablets, therefore showcasing the industrial applicability of the biosensor method herein proposed in pharmaceutical analysis.

## Conclusion

This work reports the first *S. lycocarpum* PPO-based biosensor for paracetamol detection. The biosensor was optimized regarding fruit maturation (immature and mature-ripe), as well as optimal vegetal extract volume to be used in biosensor construction as well as optimal pH of the electrochemical cell fluid. Results evidenced that the extract which rendered the biosensor with best analytical performance was from immature fruits, and the extract volume of 100 µL and neutral pH provided best detection of the phenolic probe herein used (i.e. catechol). Furthermore, the biosensor was able to quantify paracetamol in a linear range from 50 to 300 μM, showcasing LoD and LoQ of 3 μM and 10 μM, respectively. In conclusion, the biosensor herein developed may be a low-cost alternative for paracetamol determination in pharmaceutical formulations.

## Ethical Issues

Not applicable

## Conflict of Interest

Authors declare no conflict of interest in this study.
